# Supplementary material for: Characterization of Two Complete Mitochondrial Genomes of Ledrinae (Hemiptera: Cicadellidae) and Phylogenetic Analysis
Source: Insects. 2020 Sep 8;11(9):609. doi: 10.3390/insects11090609 (PMC7563726; doi:10.3390/insects11090609)
Supplement: Supplementary file 1 [file insects-11-00609-s001.pdf]

Supplementary Material

Characterization of two complete mitochondrial genomes of Ledrinae (Hemiptera: Cicadellidae) and phylogenetic analysis

Weijian Huang[1], Yalin Zhang[1]*

[1] Key Laboratory of Plant Protection Resources and Pest Management, Ministry of Education, Entomological Museum, College of Plant Protection, Northwest A&F University, Yangling, Shaanxi 712100, China; jakcyhuang@nwafu.edu.cn (W.H.)

* Correspondence: yalinzh@nwsuaf.edu.cn; Tel.: +86-029-87092190

Received: date; Accepted: date; Published: date

Table S1. Collection information of the Ledrinae species sequenced in present study.

Name	Locality	Time	Collector
Tituria sagittata	Fengxian, Shaanxi, China	22-August-2015	Wang-Xiudan
Petalocephala chlorophana	Meishan Sichuan, China	23-July-2015	Wen-Chao

Table S2. Best partitioning schemes and models based on different datasets for

Bayesian inference (BI) analysis.

Dataset	Partitioning scheme	Models
P123	P1: (*atp8*_pos2, *atp6*_pos1)	GTR+I+G
	P2: (*nad2*_pos2, *atp6*_pos2, *nad6*_pos2, *nad3*_pos2)	GTR+I+G
	P3: (*nad6*_pos3, *nad3*_pos3, *atp8*_pos3, *atp6*_pos3)	GTR+I+G
	P4: (*nad3*_pos1, *nad2*_pos1, *nad6*_pos1, *atp8*_pos1)	GTR+I+G
	P5: (*cox1*_pos1)	GTR+I+G
	P6: (*cox1*_pos2, *cox3*_pos2, *cytb*_pos2, *cox2*_pos2)	GTR+I+G
	P7: (*cox1*_pos3)	GTR+ G
	P8: (*cytb*_pos1, *cox3*_pos1, *cox2*_pos1)	GTR+I+G
	P9: (*cytb*_pos3, *cox2*_pos2, *cox3*_pos3)	GTR +G
	P10: (*nad1*_pos1, *nad4l*_pos1, *nad4*_pos1, *nad5*_pos1)	GTR+I+G
	P11: (*nad5*_pos2, *nad4*_pos2, *nad1*_pos2, *nad4L*_pos2)	GTR+I+G
	P12: (*nad1*_pos3)	GTR +G
	P13: (*nad2*_pos3)	GTR +G
	P14: (*nad5*_pos3, *nad4l*_pos3, *nad4*_pos3)	GTR +G
P123R	P1: (*atp6*_pos1)	GTR+I+G
	P2: (*cox2*_pos2, *atp6*_pos2, *cox3*_pos2, *nad3*_pos2, *cytb*_pos2)	GTR+I+G
	P3: (*nad6*_pos3, *nad3*_pos3, *atp8*_pos3, *atp6*_pos3)	GTR+I+G
	P4: (*nad2*_pos1, *nad3*_pos1, *nad6*_pos1, *atp8*_pos1)	GTR+I+G
	P5: (*atp8*_pos2, *nad6*_pos2, *nad2*_pos2)	GTR+I+G
	P6: (*cox1*_pos1)	GTR+I+G
	P7: (*cox1*_pos2)	GTR+I+G
	P8: (*cox1*_pos3)	GTR+G

	P9: (*cytb*_pos1, *cox3*_pos1, *cox2*_pos1)	GTR+I+G
	P10: (*cytb*_pos3, *cox2*_pos3, *cox3*_pos3)	GTR+G
	P11: (*nad1*_pos1, *nad4l*_pos1, *nad4*_pos1, *nad5*_pos1)	GTR+I+G
	P12: (*nad5*_pos2, *nad4*_pos2, *nad1*_pos2, *nad4L*_pos2)	GTR+I+G
	P13: (*nad1*_pos3)	GTR+G
	P14: (*nad2*_pos3)	GTR+G
	P15: (*nad5*_pos3, *nad4l*_pos3, *nad4*_pos3)	GTR+G
	P16: (*rrnS*, *rrnL*)	GTR+I+G
AA	P1: (*atp8*, *nad3*, *nad6*, *cox3*, *nad2*, *cox2*, *cytb*, *atp6*)	MTREV+I+G
	P2: (*cox1*)	MTART+I+G
	P3: (*nad4l*, *nad4*, *nad5*, *nad1*)	MTART+I+G

Table S3. Best partitioning schemes and models based on different datasets for maximum likelihood (ML) analysis.

Dataset	Partitioning scheme	Models
P123	P1: (*atp8*_pos2, *atp6*_pos1)	GTR+I+G
	P2: (*nad2*_pos2, *atp6*_pos2, *nad6*_pos2, *nad3*_pos2)	TVM+I+G
	P3: (*nad6*_pos3, *nad3*_pos3, *atp8*_pos3, *atp6*_pos3)	TRN+I+G
	P4: (*nad3*_pos1, *nad2*_pos1, *nad6*_pos1, *atp8*_pos1)	GTR+I+G
	P5: (*cox1*_pos1)	GTR+I+G
	P6: (*cox1*_pos2, *cox3*_pos2, *cytb*_pos2, *cox2*_pos2)	TVM+I+G
	P7: (*cox1*_pos3)	TIM+G
	P8: (*cytb*_pos1, *cox3*_pos1)	GTR+I+G
	P9: (*cytb*_pos3, *cox2*_pos3, *cox3*_pos3)	GTR+G
	P10: (*nad1*_pos1, *nad4l*_pos1, *nad4*_pos1, *nad5*_pos1)	GTR+I+G
	P11: (*nad5*_pos2, *nad4*_pos2, *nad1*_pos2, *nad4L*_pos2)	GTR+I+G
	P12: (*nad1*_pos3)	TIM+G
	P13: (*nad2*_pos3)	TRN+G
	P14: (*nad5*_pos3, *nad4l*_pos3, *nad4*_pos3)	K81UF+G
P123R	P1: (*atp6*_pos1)	GTR+I+G
	P2: (*cox2*_pos2, *atp6*_pos2, *cox3*_pos2, *nad3*_pos2)	TVM+I+G
	P3: (*nad6*_pos3, *nad3*_pos3, *atp8*_pos3, *atp6*_pos3)	TRN+I+G
	P4: (*nad2*_pos1, *nad3*_pos1, *nad6*_pos1, *atp8*_pos1)	GTR+I+G
	P5: (*atp8*_pos2, *nad6*_pos2, *nad2*_pos2)	TVM+I+G
	P6: (*cox1*_pos1)	GTR+I+G
	P7: (*cox1*_pos2)	TVM+I+G
	P8: (*cox1*_pos3)	TVM+G
	P9: (*cytb*_pos1, *cox3*_pos1, *cox2*_pos1)	GTR+I+G
	P10: (*cytb*_pos3, *cox2*_pos3, *cox3*_pos3)	GTR+G
	P11: (*nad1*_pos1, *nad4l*_pos1, *nad4*_pos1, *nad5*_pos1)	GTR+I+G
	P12: (*nad5*_pos2, *nad4*_pos2, *nad1*_pos2, *nad4L*_pos2)	GTR+I+G

	P13: (*nad1*_pos3)	K81UF+G
	P14: (*nad2*_pos3)	TRN+G
	P15: (*nad5*_pos3, *nad4l*_pos3, *nad4*_pos3)	K81UF+G
	P16: (*rrnS*, *rrnL*)	GTR+I+G
AA	P1: (*atp8*, *nad3*, *nad6*, *cox3*, *nad2*, *cox2*, *cytb*, *atp6*)	MTART+I+G+F
	P2: (*cox1*)	MTART+I+G
	P3: (*nad4l*, *nad4*, *nad5*, *nad1*)	MTART+I+G+F
